# The complete mitochondrial genome of *Ceresium sinicum ornaticolle* Pic, 1907 (Coleoptera: Cerambycidae)

**DOI:** 10.1080/23802359.2024.2361682

**Published:** 2024-06-17

**Authors:** Feiyi Xin, Wenlong Jiao, Yiqi Sun, Yanyue Zhou, Zhimin Zhang, Yinghua Tong

**Affiliations:** a College of Forestry, Fujian Agriculture and Forestry University, Fuzhou, China; b Key Laboratory of Integrated Pest Management in Ecological Forests, Fujian Province University, Fujian Agriculture and Forestry University, Fuzhou, China; c Minhou County Forestry Bureau, Forest Disease and Pest Control Station, Fuzhou, China; d College of Plant Protection, Fujian Agriculture and Forestry University, Fuzhou, China

**Keywords:** *Ceresium sinicum ornaticolle*, nucleotide sequences, phylogenetic tree, complete mitochondrial genome

## Abstract

*Ceresium sinicum ornaticolle* Pic, 1907 (Coleoptera: Cerambycidae) is one of the main pests of pomegranate and citrus trees. In this study, we described the complete mitochondrial genome of *C. sinicum ornaticolle*. The total length of the mitochondrial genome was 15,817 bp, and the entire content of GC was 27.8%. The genome encoded 2 ribosomal RNA genes (rRNAs), 13 protein-coding genes (PCGs), and 22 transfer RNA genes (tRNAs). The phylogenetic tree showed that *C. sinicum ornaticolle* was clustered with *Allotraeus orientalis* and *Zoodes fulguratus*. These results will provide the genetic information for understanding the genetic evolution of *C. sinicum ornaticolle* and the insights to control cerambycid pests.

## Introduction

*Ceresium sinicum ornaticolle* belongs to the subfamily Cerambycinae, a vast family of more than 25,000 species (Sama et al. 2010). The species’ body ranges from brown to dark brown, with a darker color on the head and chest that is almost entirely black. The central part of the pronotum has sparse villi, with dense villi on both sides leading to the formation of stripes. There is no significant difference in individual morphology between male and female individuals. Females produce larvae, thus causing more significant harm than males (Yiu 2009). *C. sinicum ornaticolle* is the trunk-boring pest of *Punica granatum* and *Citrus Sinensis*, as well as damage the xylem of *Cinnamomum camphora*, *Citrus* spp., and *Melia azedarach* in southern China (Lin et al. 2021). However, the information on the taxonomy and mitochondrial genome of the species is still unclear. In this study, we described the complete mitochondrial genome of *C. sinicum ornaticolle*. These results will be essential in supporting research on the future genetic evolution of *C. sinicum ornaticolle* through genetic information.

## Materials and methods

**Figure 1.**
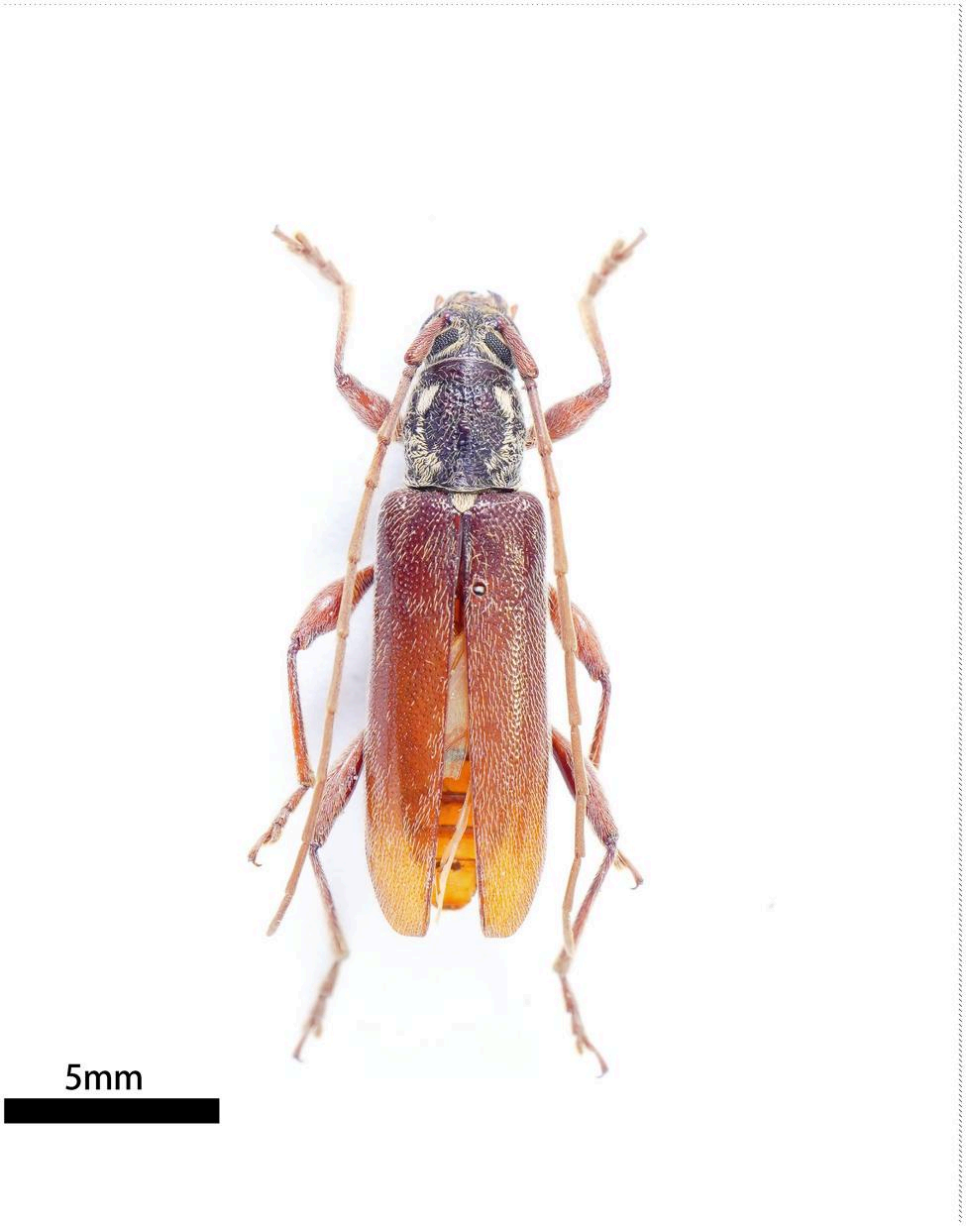
Specimen of *Ceresium sinicum ornaticolle* Pic, 1907, dorsal view. The species reference image was captured by WL Jiao.

The sample of *C. sinicum ornaticolle* was collected in Fuzhou, Fujian Province (119°23′38.18400000000012″E, 26°3′7.6320000000001″N) using the sexual trapping method. The voucher specimen (No. TN-202301) was photographed and stored in the Key Laboratory of Integrated Pest Management in Ecological Forests, Fujian Agriculture and Forestry University (contact person: Feiyi Xin; email: xinfeiyi2004@126.com) (Figure 1). We saved the remaining DNA in the above laboratory using an individual identifier (DNA-GS-202304).

In this study, the Tissue Sample Genomic DNA Extraction Kit RC1003 (Concertbio, Xiamen, China) was used to extract genomic DNA from the legs of the adult individual. The purity and concentration of the DNA were determined using NanoDrop 2000 (Thermo Fisher Scientific, USA). During library preparation, Illumina Hiseq 2500 (Illumina, San Diego, CA) was used for 2 × 150 bp paired-end sequencing (Yin et al. 2016). After filtering, 777,030 clean reads were obtained from 43,195,920 raw reads. Assembly was performed using MitoZ and metaSPAdes (Nurk et al. 2017). Subsequently, sequence annotation was conducted using the Mitos webserver (Bernt et al. 2013). To improve the annotations significantly, we used the *Omosita colon* (Nitidulidae, GenBank accession number: NC050852), which is more closely related to correction. Geneious Prime (v 2023.2) was used to visualize the mitochondrial genome map.

Phylogenetic analyses were conducted using concatenated nucleotide sequences of 13 PCGs and two rRNAs from 12 Cerambycidae species and one outgroup from Chrysomelidae. The sequence of each PCG and rRNA were aligned individually by using MUSCLE (Edgar 2022) and concatenated into a dataset. IQ-tree was used to infer phylogenetic trees using the maximum-likelihood method and estimated the optimal nucleotide substitution models (Minh et al. 2020).

## Results

**Figure 2.**
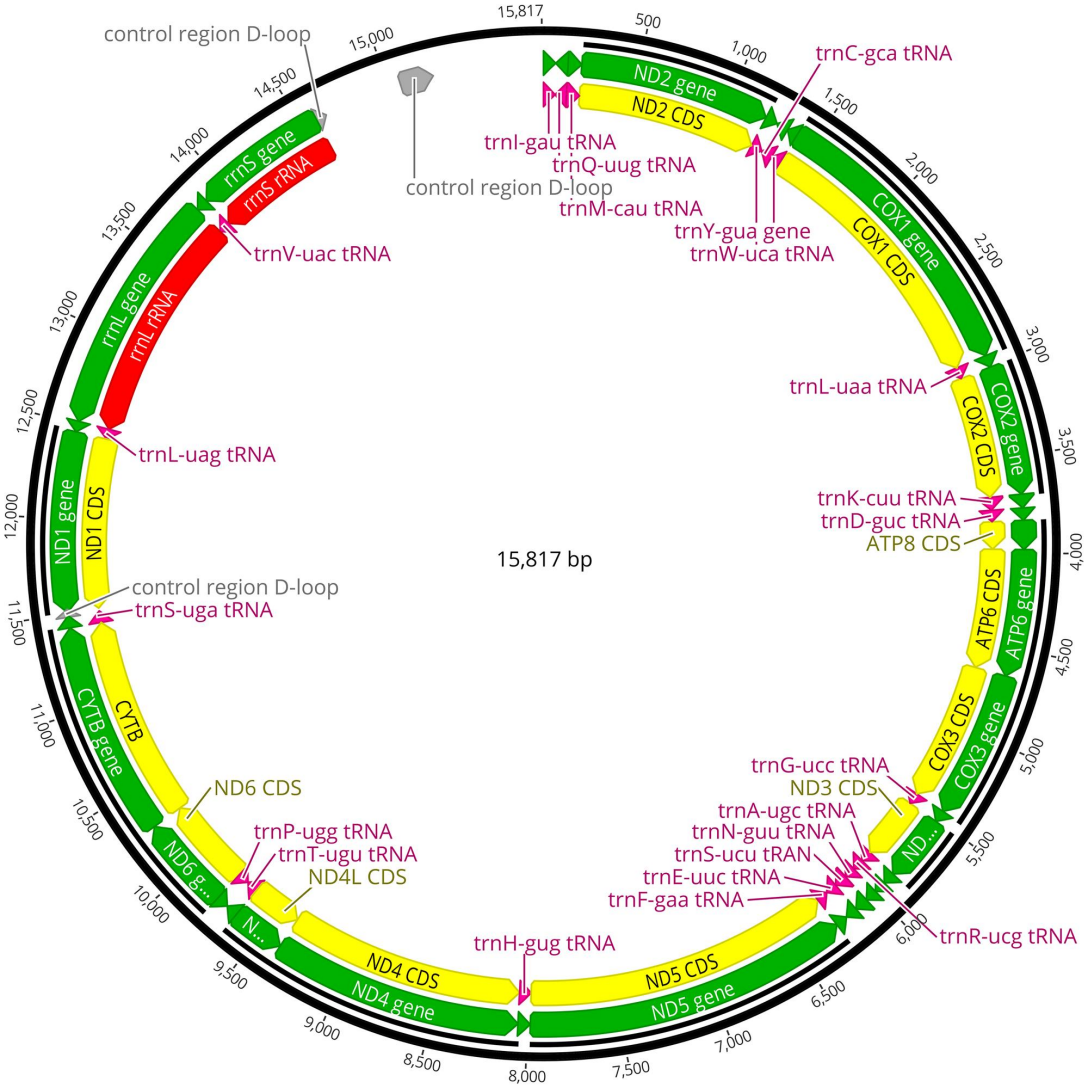
Circular map of *Ceresium sinicum ornaticolle* mitochondrial genome.

The mitochondrial genome of *C. sinicum ornaticolle* was assembled according to the depth of the coverage (average coverage of over 1000×) (Figure S1). The results showed that the complete mitochondrial genome length of *C. sinicum ornaticolle* is 15,817 bp, containing 2 ribosomal RNA genes (rRNAs) (rrnS, rrnL), 13 protein-coding genes (PCGs), and 22 transfer RNA genes (tRNAs) (Figure 2). Among these 13 PCGs, six PCGs (*ND2*, *COX2*, *ATP8*, *ND3*, *ND5*, *ND6*) started with ATT, two PCGs (*COX1*, *ATP6*) started with ATA, four PCGs (*COX3*, *ND4*, *ND4L*, *CYTB*) started with ATG, one PCG (*ND1*) started with TTG. Besides, seven PCGs (*ND2*, *COX1*, *ATP6*, *ND5*, *ND4*, *ND4L*, *ND6*) stoped with TAA, three PCG (*ATP8*, *CYTB*, *ND1*) stoped with TAG, the stop codon of the remaining three PCGs (*COX2*, *COX3*, *ND3*) was T. The entire content of GC was 27.8% (*A* = 40.1%, *T* = 32.1%, *C* = 17.0%, *G* = 10.8%) (Table S1). The mitochondrial genome sequence of *C. sinicum ornaticolle* was registered in NCBI GenBank with an accession number OR496190.

**Figure 3.**
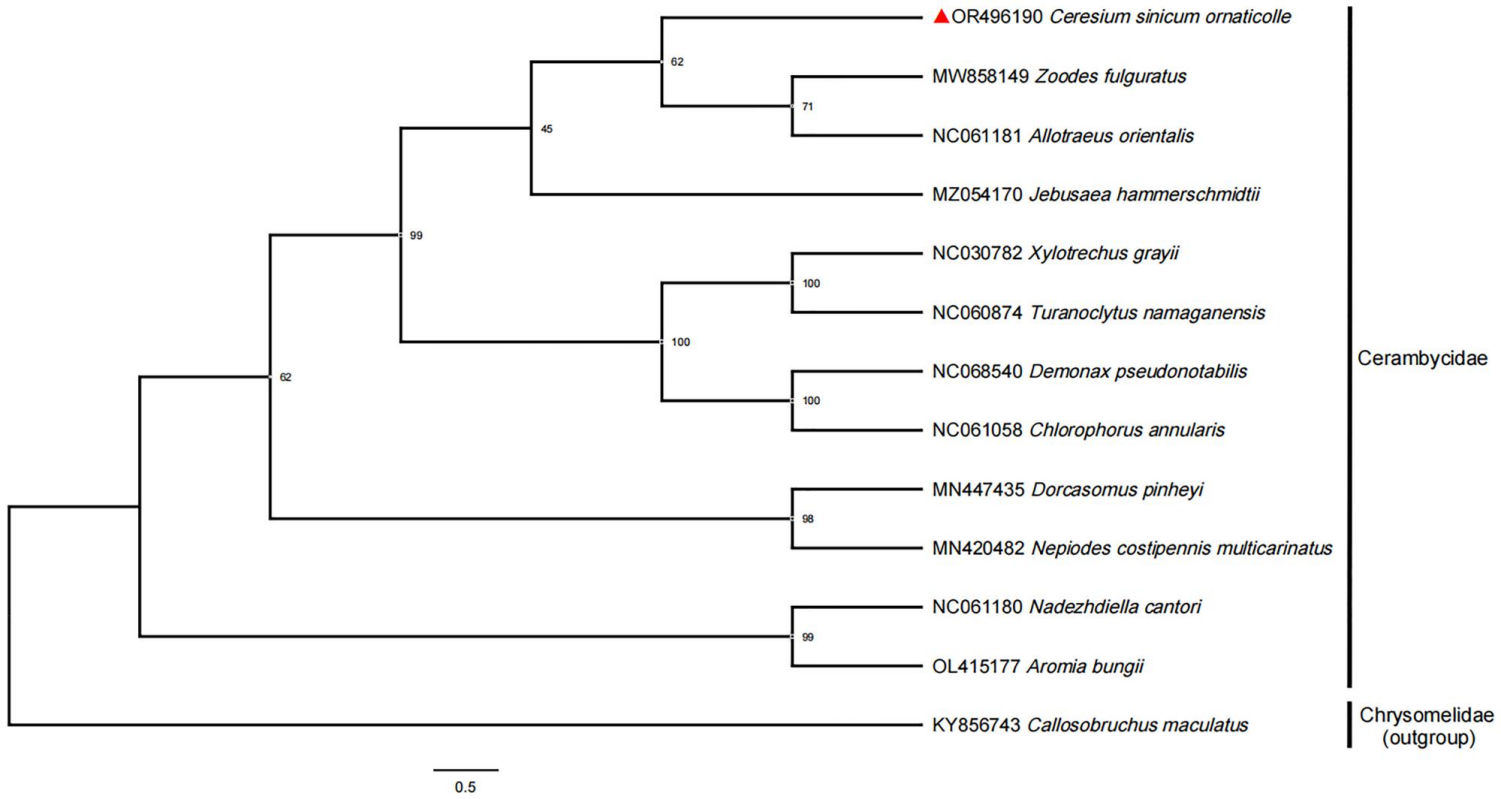
Maximum-Likelihood Phylogenetic tree of 12 different Cerambycidae species and a Chrysomelidae species based on the concatenated nucleotide sequences of 13 PCGs and two rRNAs extracted from complete mitochondrial genome. Bootstrap support values are labeled near the branch. The mitochondrial genome sequences of the different species were obtained from the GenBank databases (accession numbers are marked in the figure). The species name on the right side of the red triangle is the newly sequenced mitochondrial genome in this study (*Ceresium sinicum ornaticolle* OR496190). The following sequences were used: *Jebusaea hammerschmidti* MZ054170.1 (Dias et al. 2021), *Zoodes fulguratus* MW858149.1 (Ayivi et al. 2021), *Nadezhdiella cantori* NC_061180.1 (Tian & Li et al. 2022), *Nepiodes costipennis multicarinatus* MN420482.1 (Nie et al. 2021), *Dorcasomus pinheyi* MN447435.1 (Nie et al. 2021), *Callosobruchus maculatus* KY856743.1 (Sayadi et al. 2017) and 6 unpublished sequences (*Allotraeus orientalis* NC_061181.1, *Xylotrechus grayii* NC_030782.1, *Turanoclytus namaganensis* NC_060874.1, *Chlorophorus annularis* NC_061058.1, *Demonax pseudonotabilis* NC_068540.1, *Aromia bungi* OL415177.1).

Moreover, the phylogenetic tree showed that the *C. sinicum ornaticolle* forms a single group with the other 11 species in the family Cerambycidae and was closely related to the *Allotraeus orientalis* and *Zoodes fulguratus* (Figure 3).

## Discussion and conclusions

In this study, we sequenced and annotated the complete mitochondrial genome of *C. sinicum ornaticolle* and analyzed the overall structural characteristics. These results can serve as the basis for future research on *C. sinicum ornaticolle* and related species. The phylogenetic analysis will provide essential information for understanding the evolution of the mitochondrial genome in this species, and the mitochondrial genomic data of *C. sinicum ornaticolle* is crucial for taxonomy. It will facilitate the development of control methods for cerambycid pests, increasing yield in fruit trees and timber production.

## Data Availability

The genome sequence data supporting this study’s findings are available in GenBank of NCBI at https://www.ncbi.nlm.nih.gov under assessment no. OR496190. The associated BioProject, Bio-Sample, and SRA numbers are PRJNA1027837, SAMN37805680, and SRR26378748, respectively.
